# Determinants of neonatal mortality in Indonesia

**DOI:** 10.1186/1471-2458-8-232

**Published:** 2008-07-09

**Authors:** Christiana R Titaley, Michael J Dibley, Kingsley Agho, Christine L Roberts, John Hall

**Affiliations:** 1 School of Public Health, Edward Ford Building (A27), University of Sydney, NSW 2006, Australia; 2 George Institute for International Health, PO Box M201, Missenden Road, Sydney NSW 2050, Australia; 3 School of Medicine, University of Western Sydney, Locked Bag 1797, Penrith NSW DC1797, Australia; 4 The Kolling Institute of Medical Research, St Leonards 2065, Australia

## Abstract

**Background:**

Neonatal mortality accounts for almost 40 per cent of under-five child mortality, globally. An understanding of the factors related to neonatal mortality is important to guide the development of focused and evidence-based health interventions to prevent neonatal deaths. This study aimed to identify the determinants of neonatal mortality in Indonesia, for a nationally representative sample of births from 1997 to 2002.

**Methods:**

The data source for the analysis was the 2002–2003 Indonesia Demographic and Health Survey from which survival information of 15,952 singleton live-born infants born between 1997 and 2002 was examined. Multilevel logistic regression using a hierarchical approach was performed to analyze the factors associated with neonatal deaths, using community, socio-economic status and proximate determinants.

**Results:**

At the community level, the odds of neonatal death was significantly higher for infants from East Java (OR = 5.01, p = 0.00), and for North, Central and Southeast Sulawesi and Gorontalo combined (OR = 3.17, p = 0.03) compared to the lowest neonatal mortality regions of Bali, South Sulawesi and Jambi provinces. A progressive reduction in the odds was found as the percentage of deliveries assisted by trained delivery attendants in the cluster increased. The odds of neonatal death were higher for infants born to both mother and father who were employed (OR = 1.84, p = 0.00) and for infants born to father who were unemployed (OR = 2.99, p = 0.02). The odds were also higher for higher rank infants with a short birth interval (OR = 2.82, p = 0.00), male infants (OR = 1.49, p = 0.01), smaller than average-sized infants (OR = 2.80, p = 0.00), and infant's whose mother had a history of delivery complications (OR = 1.81, p = 0.00). Infants receiving any postnatal care were significantly protected from neonatal death (OR = 0.63, p = 0.03).

**Conclusion:**

Public health interventions directed at reducing neonatal death should address community, household and individual level factors which significantly influence neonatal mortality in Indonesia. Low birth weight and short birth interval infants as well as perinatal health services factors, such as the availability of skilled birth attendance and postnatal care utilization should be taken into account when planning the interventions to reduce neonatal mortality in Indonesia.

## Background

Of 130 million babies born annually, more than 4 million die in the neonatal period [1], and 99 per cent of these deaths occur in developing countries [2]. During the last 30 years, the reduction in neonatal mortality rates has been slower, compared to both under-five and child mortality rates after the first month of life [3,4].

According to the Convention on the Rights of the Child [5], newborns have a basic right to enjoy the highest attainable standard of health. Yet a recent review of child mortality has revealed that the proportion of under-five child deaths occurring in the first month of life has been increasing [6]. Despite accounting for almost 40 per cent of all under-five child deaths and more than half of infant deaths, neonatal mortality is not a target of the Millennium Development Goals (MDGs) [7]. However if the MDG target of a two-thirds reduction in child mortality by 2015 is to be achieved then neonatal mortality must be addressed.

Indonesia, the fourth most populous country in the world, consists of approximately 17,000 islands, and in 2001 was administratively divided into 30 provinces, 4,918 sub districts, and 70,460 villages [8]. According to the 2000 population census, the population of Indonesia reached 205.8 millions, of which 44 per cent lived in urban areas. The island of Java, which covers only seven per cent of the total area of Indonesia, is inhabited by almost 60 per cent of country's population.

In 2002 Indonesia conducted its fifth Demographic and Health Survey (DHS) of a nationally representative sample of ever-married women aged 15–49 years and currently married men aged 15–54 years. The survey aimed to gather information about child mortality, and maternal and child health, as well as family planning and other reproductive health issues.

The neonatal mortality rate in Indonesia in 2002 was reported to be 20 per 1,000 live births [8], which according to the World Health Organization estimates was similar to the average for other southeast Asian countries (19 per 1,000 live births) [9]. However over the preceding 15 years, neonatal mortality had undergone considerable improvement with a reduction in the rate of approximately 40 per cent. This reduction in neonatal mortality, as in other parts of the world, was slower than for infant, child, and under-five mortality, which fell by 48 per cent, 65 per cent and 53 per cent, respectively over the 15 year period [8,10-12].

Previous reviews of the causes of neonatal deaths have demonstrated that up to 70 per cent of neonatal mortality could be prevented using evidence-based interventions [13,14]. To adopt a focused, evidence-based approach to reduce neonatal mortality in Indonesia, a clear understanding of the associated factors is necessary. Using the 2002–2003 Indonesia Demographic and Health Survey data, this study examined the determinants of neonatal mortality for all singleton infants of the sampled women who were born between 1997 and 2002.

## Methods

### Data sources

The data examined was the 2002–2003 Indonesia Demographic and Health Survey (IDHS), which was conducted in 26 provinces, but excluded Nanggroe Aceh Darussalam, Maluku, North Maluku and Papua provinces, for security reasons.

The 2002–2003 IDHS samples for each province were stratified by urban and rural areas. Within each strata, the primary sampling unit was Census Blocks (CB) defined during the 2000 population census, which were selected using multistage stratified random sampling [8]. In urban areas, CBs were selected using systematic random sampling, followed by a random selection of twenty five households in each CB. In rural areas, the selection was done in three stages. First, sub-districts were sampled using probability proportional to the number of households in the sub-district; second, the CBs were selected using systematic sampling, and at the last stage, twenty five households were randomly selected from a list of households for each CB.

The 2002–2003 IDHS used three questionnaires, the Household Questionnaire, the Women's Questionnaire for ever-married women aged 15–49 years, and the Men's Questionnaire for all currently married men 15–54 years old. Both the Household and Women's Questionnaires were based on the standard Demographic and Health Survey Model A Questionnaires [8], designed for high contraceptive prevalence countries, and were modified to capture issues concerning family planning and health specific to Indonesia. The Household Questionnaire listed all the usual household members and recorded information about their age, sex, education and relationship to head of household as well as shared household level characteristics such as an inventory of household assets. The information recorded on the Women's Questionnaire included the women's demographic characteristics, their full birth history, their history of antenatal care for the most recent birth within a five-year period preceding the survey, delivery and postnatal care for all births, as well as the survival of their live-born infants. The information recorded on the Men's Questionnaire included their demographic characteristics and their reproductive history.

In this IDHS, 99 per cent of the 33,419 available households were successfully interviewed, and 29,483 women were interviewed which was 98 per cent of the 29,996 eligible women. Multiple pregnancies (n = 254) were excluded from this analysis, because of the substantially higher odds of neonatal death due to preterm birth and pregnancy complications compared to singleton pregnancies [15].

### Conceptual framework

**Figure 1:**
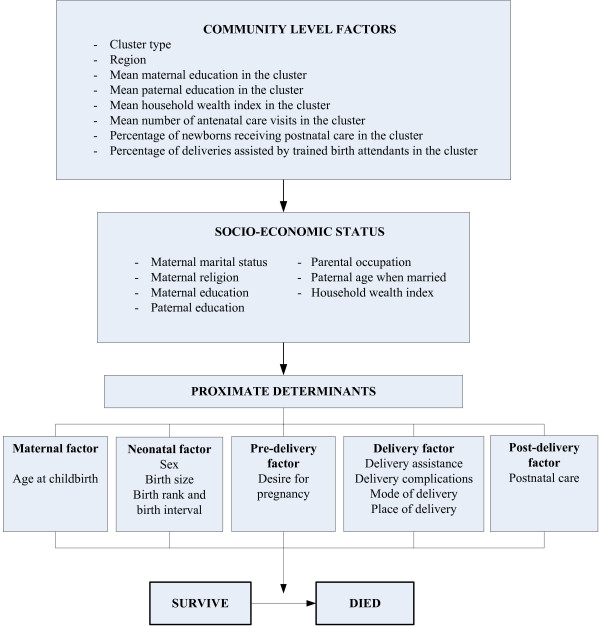
Conceptual framework for factors influencing neonatal mortality.

The Mosley and Chen conceptual framework for the study of child survival in developing countries [16] was adapted based on the available information in the 2002–2003 IDHS datasets. Figure 1 shows the framework used in this study, along with the selected possible predictors of neonatal mortality in Indonesia.

### Study variables

The primary outcome was neonatal death, which was the death of a live born infant in the first month of life. In the descriptive analyses, the neonatal mortality rate, defined as the number of neonatal deaths per 1000 live births, was used. In these analyses, the outcome was neonatal deaths recoded as a binary variable. The explanatory variables included community level contextual variables, socioeconomic and proximate determinants, covering maternal, neonatal, pre-natal, delivery, and post-natal factors (Figure 1).

Table 1 shows a list of all the variables used in this study along with their definitions and categorizations. There were eight community level (contextual) variables of which two represented the characteristics of a cluster (cluster type and region); three were socioeconomic status variables (the mean years of maternal and paternal schooling, and the mean household wealth index); and three were indicators of community access to pregnancy and delivery care services (mean number of antenatal care visits, percentage of newborns receiving postnatal care, and the percentage of deliveries assisted by trained birth attendants in the cluster). However, the variables for the average number of antenatal care visits and the percentage of newborns receiving postnatal care in the cluster only referred to the most recent delivery of a mother occurring between 1997 and 2002.

**Table 1:** Operational definition and categorization of the variables used in the analysis

**VARIABLES**	**DEFINITIONS AND CATEGORIZATION**
**COMMUNITY LEVEL FACTORS**	

Cluster type	Type of the cluster (1=urban; 2=rural)

Region	Region (1=Bali/South Sulawesi/Jambi; 2=Sumatera, excluding Jambi; 3=DKI Jakarta/West Java/Banten; 4=Central Java/DI Yogyakarta; 5=East Java; 6=NTB/NTT; 7=Kalimantan, 8=North, Central and Southeast Sulawesi and Gorontalo combined

Mean maternal education in the cluster	Mean maternal years of schooling in the cluster

Mean paternal education in the cluster	Mean paternal years of schooling in the cluster

Mean household wealth index in the cluster	Mean household wealth index for the cluster

Mean number of antenatal care visits in the cluster	Mean number of antenatal care visits in the cluster (only for the most recent birth before the survey)

Percentage of newborns receiving postnatal care in the cluster	Percentage of neonates in the cluster who received postnatal care (only for the most recent birth before the survey)

Percentage of deliveries assisted by trained birth attendants in the cluster	Percentage of births in the cluster assisted by trained birth attendants (1=<25 per cent; 2=25 per cent-49 per cent; 3=50 per cent-74 per cent; 4=75 per cent-87.4 per cent; 5=≥ 87.5 per cent)

**SOCIOECONOMIC DETERMINANTS**	

Maternal marital status	Marital status of the mother (1=currently married; 2=formerly married)

Maternal religion	Maternal religion (1=Moslem; 2=Christian; 3=other)

Maternal education	Maternal years of schooling (as continuous variable)

Paternal education	Paternal years of schooling (as continuous variable)

Parental occupation	Maternal and paternal employment status (1=mother without a job outside the home and father employed; 2=mother and father employed; 3=father unemployed)

Paternal age when married	Paternal age when married (as a continuous variable)

Household wealth index	Composite index of household amenities (as continuous variable)

**PROXIMATE DETERMINANTS**	

Maternal age at childbirth	Maternal age at childbirth (as continuous variable)

Sex	Sex of the neonate (1=female; 2=male)

Birth weight	Birth weight of the neonate (1=2500–3500 grams; 2=<2500 grams; 3=>3500 grams; 4=not weighed)

Birth size	Subjective assessment of the respondent on the birth size (1=average; 2=smaller than average; 3=larger than average)

Birth rank and birth interval	Birth rank and birth interval of neonate (1=2^nd ^or 3^rd ^birth rank, birth interval >2 years; 2=1^st ^birth rank; 3=2^nd ^or 3^rd ^birth rank, birth interval ≤ 2 years; 4=≥ 4^th ^birth rank, birth interval >2 years; 5=≥ 4^th ^birth rank, birth interval ≤ 2 years)

Desire for pregnancy	Intention to become pregnant (1=wanted then; 2=wanted later; 3=wanted no more)

Delivery assistance	Birth attendance during delivery (1=health professional; 2=traditional birth attendant/other)

Delivery complications	Complications during delivery (1=none; 2=prolonged labour; 3=other)

Mode of delivery	Mode of delivery (1 = non-Caesarean section; 2 = Caesarean section)

Place of delivery	Place of delivery (1 = home; 2 = health facility)

Postnatal care	Postnatal service received by the neonate (1 = no; 2 = yes)

The individual and household level socioeconomic variables included in the study were maternal marital status, maternal religion, maternal and paternal mean years of education, parental occupation, paternal age when married, and household wealth index. The wealth index was calculated using an inventory of households assets which were weighted using principal components analysis method [17]. The household assets used in constructing the index included; the ownership of durable goods, such as television, radio, and refrigerator; household facilities, such as electricity, and type of toilet; indicators of the condition of housing, such as the primary material used for the floor and walls; and ownership of transportation devices, such as bicycle, motorcycle and car/truck. The household wealth index was the sum of the weighted scores for each item, and was used in the analyses as a continuous variable.

Ten proximate determinants at the individual level were also identified through which the community level and socio-economic variables could possibly have had an impact on neonatal mortality. These variables were maternal age at child birth to represent maternal factors; the infant's sex, maternal subjective assessment of the infant's size, and a combined variable of infant's birth rank and birth interval which represented the neonatal factors; maternal desire for pregnancy as a pre-delivery factor; delivery assistance, delivery complications, and mode of delivery, for delivery factors; and place of delivery, as well as the postnatal care services received by neonates to represent post-delivery factors. Maternal desire for pregnancy was included in the pre-delivery factor since it might influence maternal health care and health seeking behavior during pregnancy, such as the utilization of antenatal care services. In this study, the postnatal care variable was divided into two categories, whether the infant received postnatal care services or not. During the interview, mothers were asked if their newborns ever received a postnatal check, and for the time period the first check was conducted after delivery. A neonate was considered to have received postnatal care services if this occurred within a six week (40 days) period after delivery.

### Statistical analysis

Using contingency table analyses and multilevel logistic regression the association between all possible factors and neonatal mortality was assessed.

First, frequency tabulations were conducted to describe the data used in this study, followed by the contingency table analyses to examine the impact of all potential predictors on neonatal mortality without adjusting for other covariates. All of the potential predictors were entered into the baseline model to examine their effects simultaneously.

As part of the multivariate analysis, possible associated factors were examined for evidence of collinearity, which was reflected by either the changes in the direction of effect between the univariate and multivariate analysis, or implausible standard errors for a particular variable. Once observed, the particular factor was examined against each possible factor to identify the variable with which it was highly correlated. Birth size, for example, was used in this analysis instead of birth weight, which was found to be highly correlated with delivery assistance. Furthermore, birth weight was missing for approximately 23 per cent of the infants. The average household wealth index in the cluster was also removed, because it was highly correlated to the mean number of antenatal care visits in the cluster, as well as wealth index at the household level.

Multilevel logistic regression was then performed to identify the significant independent determinants of neonatal mortality. A previously recommended [18] hierarchical approach to modeling, based on a conceptual framework describing the hierarchical relationships between different groups of variables, was applied in this analysis. In this approach the effects of more distal variables can be examined without improper adjustment by proximate or intermediate variables that may be mediators of the effects of more distal variables [18]. At the initial stage the effect of community level variables on neonatal mortality was analyzed. All the contextual variables were entered in the model, and only those that were significantly associated with neonatal mortality were retained for the subsequent model. In the second stage, the socioeconomic level variables were added to the first model, and only the significant factors were retained. The effects of the socioeconomic level variables were assessed in the presence of contextual variables. In the last stage, the proximate determinants were entered to the second model, and the effects of the significant proximate determinants were assessed in the presence of both socioeconomic and community level variables.

IDHS data sets have a hierarchical structure, with women or men within households which are within CBs. This data structure violates an underlying assumption for usual logistic regression models of independence of the observations. Instead the observations in these datasets are clustered within each CB. The multilevel modeling methods used in the analysis adjusted for this clustering of observations within CB and provided correct estimates of the standard errors

Odds ratios and 95 per cent confidence intervals were determined, and all estimates were weighted by the sampling probabilities. Two variables, maternal age at child birth and household wealth index were chosen a priori and retained in the final model, regardless of their level of significance, because they have previously been shown to be associated with the increased risk of neonatal mortality [19-21].

All of the statistical analyses were performed using STATA/MP version 10.0 (2007) (Stata Corporation, College Station, TX, USA), and the logistic regression was conducted using generalized linear latent and mixed models (gllamm) method [22,23].

## Results

To identify the associated factors for neonatal mortality, 15,952 singleton live-born infants within the five years preceding the survey were included as the study population. This analysis found that between 1997 and 2002, 54.7 per cent of infant deaths occurred during the neonatal period, of which approximately 29.9 per cent occurred on the first day and 75.6 per cent occurred in the first week of infant life.

The characteristics of the study variables are presented in Table 2 and Table 3. Approximately 89 per cent of the neonates were born to Moslem mothers and more than half of the mothers lived in rural areas. Around 57 per cent of the infants were born to mothers who did not have a job outside the home, while the fathers were employed. Only less than two per cent of infants were born to fathers who were unemployed. This study found that both the mothers and fathers had similar mean years of schooling (8 years).

**Table 2:** Characteristics of variables (n = 15952)

**VARIABLE**	**n**	n (%)*	NMR*
**COMMUNITY LEVEL FACTORS**			

**Cluster type**			

Urban	6456	6911 (46.6)	18.7
Rural	9496	7923 (53.4)	20.2

**Region**			

Bali/South Sulawesi/Jambi	1773	1036 (7.0)	9.6
Sumatera excluding Jambi	4261	3278 (22.1)	20.5
DKI Jakarta/West Java/Banten	2480	4245 (28.6)	20.2
Central Java/DI Yogyakarta	1048	1900 (12.8)	13.7
East Java	583	2067 (13.9)	25.6
NTB/NTT	1233	694 (4.7)	17.5
Kalimantan	2133	975 (6.6)	20.2
North, Central and Southeast Sulawesi combined/Gorontalo	2441	639 (4.3)	24.0

**Percentage of deliveries assisted by trained birth attendants in the cluster**			

<25 per cent	2150	2151 (14.5)	24.8
25 per cent – 49 per cent	2525	2253 (15.2)	23.0
50 per cent – 74 per cent	3184	2674 (18.0)	20.2
75 per cent – 87.4 per cent	2049	1974 (13.3)	17.2
≥ 87.5 per cent	6044	5782 (39.0)	16.6

**SOCIOECONOMIC DETERMINANTS**			

**Maternal marital status**			

Currently married	15616	15000 (97.9)	19.5
Formerly married	336	310 (2.1)	19.5

**Maternal religion**			

Moslem	13257	13000 (89.4)	20.0
Christian	1934	1287 (8.7)	13.7
Other	761	289 (1.9)	20.2

**Parental occupation**			

Mother without a job outside the home and father employed	8650	8513 (57.4)	17.1
Mother and father employed	7001	5946 (40.1)	21.7
Father unemployed	208	231 (1.6)	25.5
Missing	93	145 (1.0)	

**PROXIMATE DETERMINANTS**			

**Sex**			

Female	7720	7163 (48.3)	17.7
Male	8232	7671 (51.7)	21.2

**Birth weight**			

2500 – 3500 grams	8803	8665 (58.4)	8.4
< 2500 grams	727	723 (4.9)	72.4
> 3500 grams	2130	2104 (14.2)	6.3
Not weighed	4083	3178 (21.4)	28.4
Missing	209	165 (1.1)	

**Birth size**			

Average	8346	7843 (52.9)	12.8
Smaller than average	2193	1936 (13.1)	39.3
Larger than average	4560	4389 (29.6)	9.5
Missing	853	667 (4.5)	

**Birth rank and birth interval**			

2^nd ^or 3^rd ^birth rank, birth interval > 2 years	6024	5629 (38.0)	15.7
1^st ^birth rank	5474	5190 (35.0)	21.4
2^nd ^or 3^rd ^birth rank, birth interval ≤ 2 years	1082	977 (6.6)	24.3
≥ 4^th ^birth rank, birth interval > 2 years	2833	2579 (17.4)	18.6
≥ 4^th ^birth rank, birth interval ≤ 2 years	539	460 (3.1)	38.6

**Desire for pregnancy**			

Wanted then	13272	12000 (82.6)	16.0
Wanted later	1585	1402 (9.5)	17.3
Wanted no more	955	1054 (7.1)	18.1
Missing	140	129 (0.9)	

**Delivery assistance**			

Health professional	10551	9797 (66.0)	16.1
TBA/other	5265	4925 (33.2)	16.4
Missing	136	113 (0.8)	

**Delivery complications**			

None	10183	9370 (63.2)	15.4
Prolonged labor	3529	3307 (22.3)	12.1
Other	2017	1873 (12.6)	27.8
Missing	223	285 (1.9)	

**Mode of delivery**			

Non-Caesarean section	15375	14000 (96.1)	19.6
Caesarean section	577	581 (3.9)	16.0

**Place of delivery**			

Home	9852	8790 (59.3)	15.4
Health facility	5948	5922 (39.9)	16.2
Missing	152	123 (0.8)	

**Postnatal care**			

No	2344	1908 (12.9)	27.2
Yes	13372	13000 (85.8)	14.4
Missing	236	197 (1.3)	

*Weighted for the sampling probability

**Table 3:** Characteristics of variables (n = 15952)

**VARIABLE**	Mean* ± SE*
**COMMUNITY LEVEL FACTORS**	

Mean maternal education in the cluster	7.7 ± 0.10
Mean paternal education in the cluster	7.8 ± 0.17
Mean household wealth index in the cluster	9813.8 ± 3133.49
Mean number of antenatal care visits in the cluster	7.0 ± 0.09
Percentage of newborns receiving postnatal care in the cluster	90% ± 1.0%

**SOCIO ECONOMIC DETERMINANTS**	

Maternal education (years)	7.7 ± 0.10
Paternal education (years)	7.8 ± 0.17
Paternal age when married (years)	23.3 ± 0.15
Household wealth index	9642.0 ± 3145.72

**PROXIMATE DETERMINANTS**	

Maternal age at childbirth (years)	26.7 ± 0.10

*Weighted for the sampling probability

Approximately 59 per cent of the deliveries occurred at home, which accounted for the high percentage of neonates not weighed at birth (21 per cent). However, this survey revealed that 66 per cent of the deliveries were assisted by health professionals including doctors, nurses and midwifes. The majority (86 per cent) of the babies received postnatal care services and 83 per cent (95% CI: 81.6–84.3) received them during their first week of life (0–7 days).

A close relationship was also observed between the utilization of different perinatal health care services, such as antenatal checks, postnatal care and delivery assistance. The infants who received at least four antenatal checks were also more likely to have attended postnatal care services (91 per cent, 95% CI: 90.0–92.1) and to have been delivered by health professionals (75 per cent, 95% CI: 72.8–77.7). Similarly, approximately 94 per cent (95% CI: 92.5–94.4) of infants delivered by health professionals also received postnatal care services, compared to only 76 per cent (95% CI: 73.1–78.4) for infants delivered by untrained attendants.

Table 4 summarizes the crude and adjusted odds ratios of the possible factors associated with neonatal mortality. This study found a wide variation in the odds of neonatal mortality by province. The provinces of Bali, South Sulawesi and Jambi were combined and selected as the reference group due to their low neonatal mortality rates based on the 2002–2003 IDHS report, which were 9, 12, and 14 per 1000 live births, respectively [8]. When individual provinces were entered in the final model, a higher odds of neonatal death was observed in East Java (OR = 4.92, 95% CI: 1.96–12.37, p = 0.00), Southeast Sulawesi (OR = 3.99, 95% CI: 1.09–14.59, p = 0.04), Lampung (OR = 3.67, 95% CI: 1.28–10.55, p = 0.02) and South Sumatera (OR = 3.40, 95% CI: 1.03–11.30, p = 0.05), compared to Bali/South Sulawesi/Jambi provinces combined. In the final multivariable model, groups of provinces (region) were used and revealed that the odds of neonatal death was significantly higher in East Java (OR = 5.01, p = 0.00) and North, Central and Southeast Sulawesi and Gorontalo combined (OR = 3.17, p = 0.03) than in Bali, South Sulawesi and Jambi provinces combined.

**Table 4:** Factors associated with neonatal mortality: unadjusted and adjusted odds ratio

**VARIABLE**	**Unadjusted**				Adjusted*			
		
	**OR**	**95% CI**		***p***	**OR**	**95% CI**		***p***
**COMMUNITY LEVEL**								

**Cluster type**								

Urban	1.00							
Rural	1.34	1.03	1.72	0.03				

**Region**								

Bali/South Sulawesi/Jambi	1.00				1.00			
Sumatera excl. Jambi	1.98	0.99	3.98	0.05	2.31	0.91	5.86	0.08
DKI Jakarta/West Java/Banten	1.89	0.95	3.75	0.07	2.17	0.87	5.39	0.10
Central Java/DI Yogyakarta	1.54	0.73	3.27	0.26	2.36	0.91	6.14	0.08
East Java	2.64	1.30	5.35	0.01	5.01	2.00	12.59	0.00
NTB/NTT	1.59	0.64	3.91	0.32	1.16	0.34	4.00	0.81
Kalimantan	2.01	0.9	4.47	0.09	1.87	0.63	5.54	0.26
North, Central and Southeast Sulawesi/Gorontalo	2.57	1.12	5.92	0.03	3.17	1.11	9.02	0.03

**Mean maternal education in the cluster**	0.94	0.89	0.99	0.01				

**Mean paternal education in the cluster**	0.98	0.95	1.02	0.29				

**Mean household wealth index in the cluster**	0.99	0.99	0.99	0.01				

**Mean number of antenatal care visits in the cluster**	0.90	0.85	0.95	0.00				

**Percentage of newborns receiving postnatal care in the cluster**	0.35	0.18	0.69	0.00				

**Percentage of deliveries assisted by trained birth attendants in the cluster**								

<25 per cent	1.00				1.00			
25 per cent – 49 per cent	0.89	0.60	1.32	0.57	1.03	0.65	1.65	0.90
50 per cent – 74 per cent	0.79	0.54	1.17	0.24	0.78	0.48	1.26	0.31
75 per cent – 87.4 per cent	0.53	0.33	0.84	0.01	0.47	0.26	0.84	0.01
≥ 87.5 per cent	0.51	0.36	0.73	0.00	0.40	0.25	0.63	0.00

**SOCIO ECONOMIC DETERMINANTS**								

**Maternal marital status**								

Currently married	1.00							
Formerly married	1.00	0.42	2.36	0.99				

**Maternal religion**								

Moslem	1.00							
Christian	0.53	0.30	0.95	0.03				
Other	1.13	0.49	2.62	0.78				

**Maternal education**	0.97	0.94	1.00	0.07				

**Paternal education**	1.01	0.98	1.05	0.35				

**Parental occupation**								

Mother without a job outside the home and father employed	1.00				1.00			
Mother and father employed	1.47	1.14	1.89	0.00	1.84	1.35	2.50	0.00
Father unemployed	1.57	0.64	3.89	0.33	2.99	1.18	7.55	0.02

**Paternal age when married**	1.01	0.98	1.04	0.52				

**Household wealth index**	0.99	0.99	0.99	0.01	0.99	0.99	1.00	0.61

**PROXIMATE DETERMINANTS**								

**Maternal age at child birth**	0.98	0.97	1.00	0.14	0.99	0.96	1.02	0.48

**Sex**								

Female	1.00				1.00			
Male	1.25	0.98	1.61	0.08	1.49	1.10	2.02	0.01

**Birth weight**								

2500 – 3500 grams	1.00							
< 2500 grams	6.27	4.15	9.46	0.00				
> 3500 grams	0.75	0.42	1.36	0.35				
Not weighed	3.32	2.41	4.57	0.00				

**Birth size**								

Average	1.00				1.00			
Smaller than average	2.96	2.13	4.11	0.00	2.80	1.98	3.98	0.00
Larger than average	0.81	0.55	1.18	0.26	0.85	0.58	1.25	0.41

**Birth rank and birth interval**								

2^nd ^or 3^rd ^birth rank, birth interval >2 yrs	1.00				1.00			
1^st ^birth rank	1.48	1.09	2.00	0.01	1.21	0.79	1.83	0.38
2^nd ^or 3^rd ^birth rank, birth interval ≤ 2 yrs	1.89	1.18	3.02	0.01	1.90	1.07	3.38	0.03
≥ 4^th ^birth rank, birth interval >2 yrs	1.32	0.91	1.93	0.15	1.12	0.67	1.87	0.68
≥ 4^th ^birth rank, birth interval ≤ 2 yrs	3.11	1.84	5.26	0.00	2.82	1.46	5.46	0.00

**Desire for pregnancy**								

Wanted then	1.00							
Wanted later	1.18	0.76	1.84	0.47				
Wanted no more	1.30	0.80	2.11	0.29				

**Delivery assistance**								

Health professional	1.00							
TBA/other	1.21	0.91	1.60	0.20				

**Delivery complications**								

None	1.00				1.00			
Prolonged labor	0.80	0.55	1.18	0.26	0.70	0.46	1.07	0.10
Other	2.04	1.45	2.86	0.00	1.81	1.25	2.63	0.00

**Mode of delivery**								

Non-Caesarean section	1.00							
Caesarean section	0.95	0.49	1.82	0.88				

**Place of delivery**								

Home	1.00							
Health facility	0.90	0.68	1.20	0.48				

**Postnatal care**								

No	1.00				1.00			
Yes	0.48	0.34	0.67	0.00	0.63	0.42	0.95	0.03

* 1216 missing cases were excluded from the analysis

The odds of neonatal death were 81 per cent higher for neonates born to mothers experiencing complications during delivery, such as vaginal bleeding, fever, and convulsions. For newborns, whose birth size according to the mother was smaller than average, the odds of dying was approximately 2.8 times the odds for average-sized babies. When the variable for infants' size was replaced by the birth weight variable in the final model, birth weight remained as a strong predictor, with the odds for neonatal death for low birth weight infants (<2500 grams) was 5.5 times (95% CI: 3.59–8.57, p = 0.00) the odds for the normal weight infants (2500 – 3500 grams).

Another important predictor for neonatal mortality was the combined parental employment status. Compared to infants born to fathers who were employed and mothers who did not have a job outside the home, the odds of dying was significantly higher for infants whose both parents were employed (p = 1.84, p = 0.00) and for infants whose fathers were unemployed (OR = 2.99, p = 0.02) (Table 4).

Health care service factors demonstrated a significant association with neonatal mortality. As seen in Table 4, there was a progressive reduction in the odds of neonatal death as the percentage of deliveries assisted by trained birth attendants in the cluster increased. For those infants born in clusters where ≥ 87.5 per cent of deliveries were assisted by trained birth attendants, there was a 60 per cent reduction in neonatal deaths compared to clusters with <25 per cent deliveries assisted by trained birth attendants. When this variable was replaced by other cluster level indicators of perinatal health services, the mean number of antenatal care visits in the cluster or the percentage of newborns receiving postnatal care in the cluster, the odds of neonatal deaths decreased significantly in the cluster with a higher mean number of antenatal care visits (OR = 0.86, 95% CI: 0.80–0.92, p = 0.00) or a higher percentage of postnatal care attendance (OR = 0.14, 95% CI: 0.06–0.32, p = 0.00). These cluster level perinatal health services indicators were not entered into the model simultaneously as they were found to be highly correlated to each other or to other perinatal health services indicators at the individual level, such as the postnatal care services. At the individual level there was also a significant reduction in the odds of neonatal death of 37 per cent for neonates who received postnatal care checks, compared to those without any health care after birth.

There was a significant 49 per cent increased odds of neonatal death for males compared with females. The mortality odds for fourth or higher birth rank neonates with a short preceding birth interval (less than or equal to two years) was more than 2.8 times the odds for second or third rank infants with a longer birth interval. A short birth interval of the second or the third rank infants also showed an increased odds of neonatal deaths (OR = 1.90, p = 0.03).

## Discussion

Our analyses of the 2002–2003 IDHS have revealed that the availability and use of perinatal health care services were associated with reduced odds of neonatal deaths. Clusters with a higher percentage of deliveries assisted by trained birth attendants, and the individual utilization of postnatal care services were significantly associated with reduced odds of neonatal deaths. However, we also found a variety of factors that significantly increased the odds of neonatal death. Ordered from the most significant odds, the proximate determinants associated with neonatal deaths were high birth rank and short birth interval infants, smaller than average-sized infants, complications during delivery, and male infants. The socioeconomic factors found to be associated with neonatal deaths were maternal occupation outside the home and paternal unemployment. At the community level, the regions of East Java and North, Central and Southeast Sulawesi and Gorontalo combined were found to be highly associated with increased odds of neonatal death. A progressive reduction in the neonatal death odds was observed as the percentage of deliveries assisted by trained birth attendants in the cluster increased. The identification of these determinants of neonatal deaths is important to provide guidance for the development of evidence-based approaches directed at reducing neonatal mortality in Indonesia.

This study had several strengths. First, the 2002–2003 IDHS was a nationally representative survey, using standardized methods that achieved high individual (98 per cent) and household (99 per cent) response rates. The second was the use of infant survival data from a five-year period preceding the survey which has been shown to reduce recall errors about birth and death dates by the interviewed mothers [4,24,25]. The third was the use of the random effect multilevel modeling that took into account the hierarchical structure of the data as well as the variability within the community, household and individual levels to better estimate the level of association of the study factors with the outcome [26].

However, the study had several limitations that should be noted when interpreting the results. First, only surviving women were interviewed, which may have lead to an underestimate of the neonatal mortality rate, because of the association of neonatal deaths with maternal deaths. This could also have lead to an underestimate of the effect of some of the associated factors, such as delivery complications [24]. Second, there are other possible determinants of neonatal mortality which were not available in the IDHS dataset, such as environmental and genetic factors, or were only available for the most recent delivery of a mother occurring within the last five years preceding the survey, such as the utilization of antenatal care services. Third, several variables in the study were not infant-specific because they only reflected the most recent conditions or birth, such as maternal and paternal occupation, which represented the employment status within the last twelve months preceding the survey. Fourth, the initiation of breastfeeding variable was also omitted in this analysis due to the low number of neonatal deaths in the late neonatal period (7–28 days), which was hypothesized as the time when colostrum would start to provide protection to the infant for infectious diseases.

Smaller infant size emerged as one of the strongest predictors of neonatal mortality. When it was replaced by the birth weight variable in the final model, there was a consistency of effect demonstrating the significant influence of birth weight on neonatal death. This finding is supported by other literatures that have identified low birth weight as a strong predictor of neonatal mortality [2,27]. A study in Bangladesh reported that approximately 75 per cent of neonatal deaths associated with low birth weight were attributed to preterm birth rather than small for gestational age infants [28]. However, in this study, we were unable to differentiate between preterm and small for gestational age infants.

The results of this study also indicated that neonates born to women experiencing complications such as vaginal bleeding, fever or convulsions during childbirth had remarkably higher odds of dying compared to those born to women without any complications. A study in Bangladesh revealed that infants born to women without severe delivery complications had better survival than those born to women with ecclampsia, intra-partum hemorrhage, or even prolonged labor [29]. Appropriate antenatal care can play a role by educating women and their families to recognize delivery complications that require referral to health care services to achieve a better health outcome for both mothers and infants.

The sex of the neonates significantly influenced the odds of dying, and consistent with other reports we found females had a lower odds of mortality than males during the first month of life [30-34]. This increased odds may be also due to the large proportions of neonatal deaths occurring in the first week, which is the time when gender differences in neonatal mortality are more pronounced [33]. The biological factors that have been implicated with this increased risks of neonatal deaths in male infants include immunodeficiency [32] increasing the risks of infectious diseases in males, late maturity [33] resulting in a high prevalence of respiratory diseases in males, and congenital malformations of the urogenital system.

Strong associations have previously been reported between short preceding birth interval, birth rank and the risks of neonatal death [35-37]. Similarly in this analysis, the combination of a higher birth rank and a shorter interval provided a higher odds than lower rank with a shorter birth interval. This could be related to maternal depletion syndrome and resource competition between siblings, in addition to a lack of care and attention experienced by high-ranked infants [35,38].

As an indicator of health care service utilization after delivery, postnatal care services received by the infants showed a significant protective effect. This result demonstrated an important role of postnatal care services in reducing neonatal mortality in Indonesia. Different kinds of postnatal care interventions should be carried out which have been proven to be effective to prevent neonatal deaths, including facility based, population outreach, or family-community based interventions [13,39,40]. In Indonesia, each newborn is recommended to receive at least two basic-health care checks within the periods of 0–7 days and 8–28 days, to examine the newborn for illness and provide information on appropriate infant care to the mothers. The national coverage of postnatal care checks in Indonesia was reported to be less then 66 per cent in 2005 and approximately 25 per cent of the provinces had below this average national level [41]. These results suggest the need for public health interventions directed at improving the awareness of mothers and family members about the importance of postnatal care checks and to further increase the utilization of these services. Quality, accessibility, and availability of the services should be enhanced to ensure optimal results for neonatal health.

The combined parental occupation in this analysis was significantly associated with neonatal death even after controlling for household wealth index. Paternal unemployment and maternal occupation outside the home significantly increased the odds of neonatal death. Paternal employment would have had a protective effect on neonatal mortality by increasing household income and economic status, while maternal participation in the labor force may have adversely affected the care provided to the newborn. Lack of personal and timely care including infrequent breastfeeding experienced by infants born to working mothers have been reported to increase the odds of neonatal death [37]. However, it should be noted that parental employment status in the IDHS only reflected parental participation in the labor force during the year before the survey. Nevertheless, this finding suggests that ensuring adequate maternal care during the first month of life is critical, especially for infants born to working mothers.

At the community level, the region where the mother lived and the percentage of deliveries assisted by trained birth attendants had a significant influence on neonatal death. The presence of skilled birth attendants, either medical doctors, nurses, or midwives, is important to ensure appropriate management of the delivery process and prevent fatal events attributed to delivery-related complications [1]. These findings indicate the importance of ensuring skilled birth attendance is available at the community level. Furthermore, efforts to enhance the skills of birth attendants will also contribute to reducing neonatal mortality.

## Conclusion

The 2002–2003 IDHS data examined in this analysis demonstrated that individual, household and community level variables had a significant impact on neonatal mortality. These findings point to the need for comprehensive prevention strategies to further reduce neonatal mortality in Indonesia.

At the community level, particularly in regions where the odds of neonatal death is significantly higher, the quality of the health infrastructure as well as the availability of skilled birth attendants will have significant impact in reducing neonatal mortality. At the household and individual levels, health promotion strategies to increase awareness of the importance of timely and appropriate postnatal care service utilization and the benefits of birth spacing are needed given their protective effect on neonatal mortality. Interventions to prevent low birth weight would also contribute to further reductions of neonatal mortality in Indonesia.

## Competing interests

The authors declare that they have no competing interests.

## Authors' contributions

CRT and MJD designed the study; CRT performed the analysis and prepared the manuscript; MJD, KA, CLR and JH provided data analysis advice, and revision of the final manuscript. All authors read and approved the manuscript.

## Pre-publication history

The pre-publication history for this paper can be accessed here:


